# Influence of Negative Emotional Expressions on the Outcomes of Shared Decision Making During Oncofertility Consultations in Japan

**DOI:** 10.1089/jayao.2017.0108

**Published:** 2018-08-01

**Authors:** Tomoe Koizumi, Kazuko Nara, Tomoko Hashimoto, Satoru Takamizawa, Kouhei Sugimoto, Nao Suzuki, Yoshiharu Morimoto

**Affiliations:** ^1^ Researcher to Deputy Director, National Research Institute for Child Health and Development, NCCHD, Tokyo, Japan.; ^2^ Department of Clinical Psychology, Kameda Medical Center, Kamogawa, Japan.; ^3^ Division of Integrated Medicine, IVF Namba Clinic, Osaka, Japan.; ^4^ Reproduction Center, International University of Health and Welfare Hospital, Nasushiobara, Japan.; ^5^ Department of Obstetrics & Gynecology, Dokkyo Medical University Koshigaya Hospital, Koshigaya, Japan.; ^6^ Department of Obstetrics and Gynecology, St. Marianna University School of Medicine, Kanagawa, Japan.; ^7^ HORAC Grand Front Osaka Clinic, Osaka, Japan.

**Keywords:** decisional conflict, negative emotional evaluation, oncofertility consultation, patient–family–doctor communication, shared decision making

## Abstract

This report examines how negative emotional expressions (NEE) influence the consequences of shared decision making (SDM) in oncofertility treatment among 32 young female cancer patients and 19 family members. Using a cross-sectional observational study, results showed that NEE influence the outcome consequences of SDM related to patients' decisions about desired treatment(s) and that the absence of negative emotional reactions to information from doctors was related to willingness to receive the desired treatment. This suggests that healthcare providers need to be sensitive to NEE of patients and their families, and highlights the need for psychological counseling before oncofertility consultation.

## Introduction

Many young adult female cancer patients have various fertility concerns before cancer treatment, including fertility preservation (FP),^1^ chemotherapy-induced amenorrhea, premenstrual ovarian insufficiency, and menopause symptoms during/after cancer treatment.^2^ According to the guidelines of the American Society of Clinical Oncology^3^ and the American Society for Reproductive Medicine,^4^ healthcare providers interview patients about fertility concerns to inform reproductive doctors and psychological counselors.^5,6^ There are organizations in the fields of oncology, obstetrics, and gynecology in different countries. In Japan, certified oncofertility psychologists with specialized skills were approved in 2016.^6^ These professionals support the fertility concerns of female cancer patients.

Patients' decision making on FP and/or fertility treatment is influenced by medical, social, and psychological dynamics. First, there are cases where doctors do not agree with the medical intervention based on medical guidelines, even when the patients prefer otherwise.^7,8^ This may be because such doctors think that treatment may not lead to a positive clinical outcome. Medical factors therefore influence decision making about fertility treatment among cancer survivors.^9^ Second, cost and public insurance may affect decision making in terms of implementing FP.^10^ Psychological factors, including mental illness and decisional conflict, can also affect decision making about fertility treatment. Cancer diagnosis might lead to severe shock and a sense of hopelessness, major depression^11^ and anxiety disorders, such as post traumatic stress disorder (PTSD).^12^ Depressive and/or PTSD symptoms may cause some patients to decline treatment even when it is recommended by doctors.^13^ Decisional conflict thus increases due to unmet information needs^2^ and can also result from bad patient–doctor communication and little support during decision making.^10^

Recently, the role of emotion in decision making has received some attention in extant literature.^14–16^ For instance, cancer patients' negative emotions during consultation influence their decisional conflict and anxiety through shared decision making (SDM).^17^ It was recently reported that patients and caregivers in an Asian oncology setting commonly expressed negative emotions.^18^ However, there are few studies on the direct effect of negative emotional expressions (NEE) on the consequences of SDM at the OncoFertility Consultation (OFC). This study therefore investigates whether patients' emotions associated with patient–doctor communication during fertility specialists' medical consultations are related to the consequences of shared decision making. It also examines how patients' NEE and that of their family members affects SDM.

## Methods

This was an observational, cross-sectional study involving a mixed method of quantitative and qualitative data. Thirty-three young adult female cancer patients, aged between 20 and 45 years, were recruited into the study. The patients desired FP, fertility treatment, transplantation of ovarian tissue cryopreservation or frozen embryo transfer, and came to the OFC of the Obstetrics and Gynecology Departments of two college hospitals in the Tokyo metropolitan area with some of their family members. The study period was from June 2014 to October 2014.

Five doctors were in charge of outpatient oncofertility treatment. They documented the reasons for patient consultations and the desired treatment, and also provided relevant medical information, data on realizable oncofertility treatments, and patients' or families' wishes and views.

### Ethical approval

All the participants gave their informed consent to the study.

### Data collection

Nine psychologists (certified in clinical and reproductive psychology), who had at least 5 years of clinical experience each, and no prior professional relationships with any of the patients, collected the data. To eliminate observational bias, they were forbidden to collect personal information from patients and their families, or to talk with them. They sat off to the side of the consultation room to observe the behaviors of doctors, patients, and families, and recorded the following items: attributes, status of cancer diagnosis and treatment, attendant on the day, the number of OFC, the purpose of OFC, the patient's psychological assessment, the family's psychological assessment, the patient's comprehension level, and the process of SDM. All data were made anonymous and pseudonyms were used.

It is important to note that previous behavioral studies have used methods such as the Rotar Interaction Analysis System^19^ and reminiscence methods. However, these have been associated with impression and recall biases, respectively.^20,21^

### Data analyses

Because a patient was hospitalized before the consultation, she and her attending family member were excluded. There were 32 patients and 19 attending families.

All data obtained were classified into three types of SDM at the end of OFC (Type 1: “realized group” comprising patients who receive treatment according to the patient's wishes, Type 2: “unconcluded group” comprising patients who did not reach a decision during consultation, Type 3: “unrealized group” comprising patients who could not receive treatment according to the patient's wishes).

Coding was done based on the definition of Nakatani.^22^ The frequency was calculated for each person, converted into quantitative data, and taken as the NEE score. Whether the NEE was related to SDM was determined using chi-square. A two-digit place in NEE of patients was taken from the distribution of the score; 0, 1 (*n* = 16), and 2 or more (*n* = 16) were classified as the high and low groups, respectively. The issue of whether the family member expressed negative emotions (*n* = 9) or not (*n* = 10) was categorized into two groups. The independent test using Fisher's exact test was performed using crosstabulation with NEE (two categories) × SDM (three categories).

## Results

### Characteristics by three SDM types

Using one-way ANOVA, the SDM types of the subjects did not show significant differences according to the patient's age on the day of consultation and their age at the onset of cancer (*F* (2, 29) = 2.504, *p* = 0.099; *F* (2, 27) = 2.991, *p* = 0.067). The percentage of married patients was significant (*χ*^2^ (2) = 7.748, *p* = 0.017, Cramer's V = 0.488). There were more married patients in the realized group (adjusted residuals 2.6) and more unmarried patients in the Type 2 group in the residual analysis (adjusted residuals 2.5). Also, there was a significant relationship between “whether patients promised to continue clinical visits after the next time or not,” and significantly more “promise to continue patients” in Type 1 (adjusted residual 1.9). More “no promise to continue patients” existed in Type 3 (adjusted residual 4.3) in residual analysis. Patients' understanding of medical information was not significant (*χ*^2^ (2) = 3.889, *p* = 0.108, Cramer's V = 0.365)

### Relationship between patients' NEE and the SDM types

Statements concerning the expression of negative emotions by patients and their families showed that 27 patients (84.4%) made at least one such statement, whereas five patients (15.6%) made none. Nine out of the 19 family members (47.4%) made one such statement, whereas 10 (52.6%) made none.

The chi-square was significant and signified that patients who expressed many negative emotions were more likely to be in Type 3 (adjusted residual 2.4) (Table 1).

**Table 1.** Relationship Between Patients' Negative Emotional Expressions and Shared Decision Making

*SDM*	*Type 1*	*Type 2*	*Type 3*	*Total*
Patients' NEE				
0–1	8	8	0	16
2–4	4	7	5	16
Total	12	15	5	32

*χ*^2^(2) = 6.241, *p* = 0.050, Cramer's V = 0.447.

NEE, negative emotional expressions; SDM, shared decision making.

### Relationship between patients' negative emotional reactions to information provided by doctors and the SDM types

There was a significant difference between negative expressions as reactions and SDM types, and the effect size was large. Patients who had no negative emotional reaction to medical explanations by the doctor were likely to be in Type 1 in residual analysis (adjusted residual 2.9) (Table 2).

**Table 2.** Relationship Between Presence or Absence of Patients' Negative Emotional Reactions to Medical Information from Doctors and Shared Decision Making

*SDM*	*Type 1*	*Type 2*	*Type 3*	*Total*
Patients' negative emotional reactions				
Absence	10	5	1	16
Presence	2	10	4	16
Total	12	15	5	32

*χ*^2^(2) = 8.576, *p* = 0.012, Cramer's V = 0.524.

### Relationship between patient and family NEE

Family members' NEE was not significantly related to age, marital status, initial consultation, cancer treatment, or the purpose of the consultation. However, there was a significant difference between NEE of patients and that of their family members (*χ*^2^ (1) = 11.825, *p* = 0.001, φ coefficient = 0.789). Families tended to show less NEE when patients showed less, (adjusted residual 3.4), and more NEE when patients showed more.

### NEE of the family member and the SDM types

There was a significant relationship between families' NEE and the SDM types, and the effect size was large. When families did not express negative emotions, patients were more likely to be in Type 1 (adjusted residual 2.2), and when families expressed negative emotions, patients were more likely to be in Type 3 (adjusted residual 2.4). The result is shown in (Table 3).

**Table 3.** Relationship Between Families' Negative Emotional Expressions and Shared Decision Making

*SDM*	*Type 1*	*Type 2*	*Type 3*	*Total*
Families' NEE				
Absence	6	4	0	10
Presence	1	4	4	9
Total	7	8	4	19

*χ*^2^(2) = 7.114, *p* = 0.024, Cramer's V = 0.630.

### Relationship between attendant families' negative emotional responses to information provided by the doctor and the SDM types

Among the family members, 17 had no response, whereas two had reactions. However, there was no significant relationship between the presence or absence of reactions and the conclusion on the consultation date.

## Discussion

According to behavioral observations during obstetrics and gynecologist–patient communications, the results of this study showed that the NEE of patients and attending families during consultation were related to the consequences of SDM. They also showed that patients express negative emotions as reactions to medical information provided by doctors. These were linked to feelings of threat and anxiety, which overlapped with other mental shocks, such as a cancer diagnosis or severe cancer treatment, loss of fertility, and reproductive preservation, as shown in the results.

The dual process theory may help explain how the feelings of patients and their families relate to SDM in OFC. Along these lines, it is possible that self-control based on thought (termed “system 2”) did not lead to decision making when added to cognitive processing for multiple, complicated tasks. This could have switched decision making toward automatic deduction through intuition and emotion that occurred in the moment. This is termed “system 1”.^15–17^ Many cancer patients have existing emotional shock and anxiety before OFC.^23^ When patients felt anxious due to cancer diagnosis and treatment, they were likely to make decisions using system 1. This would also apply to their partners or family members, who would probably make intuitive decisions in the moment.

That a patient's understanding of medical information alone does not influence SDM suggests the need for a patient-centered decision aid. A previous study reported that when doctors convey optimistic thoughts to patients, they stopped their negative expressions,^24^ but felt more anxious after consultation when doctors expressed consensus and more sympathy.^10^ Although healthcare providers may think that comforting patients is good, it is more important to listen carefully without obstructing the patients' narratives.

This study found that negative feelings were often spontaneously elicited as anxiety and shock in response to information provided by physicians during consultation. This suggests the need for healthcare providers to attend to the emotions of patients and their families; for instance, by listening to them during cancer consultation treatment. This may help loosen tension and create a sense of calm. Clinical psychologists should do counseling to assess psychological phases and alleviate distress, while healthcare providers should ask about their values and preferences before consultation, which will smooth communication and foster stable emotions. Incorporating a multidisciplinary medical staff may also help patients to reach useful outcomes through SDM. Lastly, in Japan, sociocultural characteristics shape decision making in cancer treatment, and families' willingness is often important.^25^ This should provide a suitable background for SDM to flourish in oncofertility consultations.

There are a number of limitations associated with this study: First, the small number of subjects. Second, evaluations were conducted on the spot because behavioral observations cannot be recorded due to the rules of clinical practice. Although it was good that the psychologist could evaluate emotional expressions in the context of communication as a third party, a conversation analysis between the three could take place to clarify the context. Also, collecting data from both patient and family conversations can help clarify the relationship between feelings and decisions, if a detailed behavioral observation was carried out. Another limitation of the study relates to its inability to evaluate whether the fertility decisions made through SDM remain unaltered throughout the treatment period. Future and similar studies must therefore take these limitations into consideration, improve the accuracy of the evaluations, and conduct international comparison studies to replicate and test the generalizability of the study's findings.

## Conclusion

The NEE of Japanese patients and their families during OFC may influence the outcomes of SDM. Since depression is often associated with cancer diagnosis and cancer treatment, psychological counseling before consultation may eliminate tension, foster calm, and relieve stress. To this end, a multidisciplinary clinical staff may help complement missing skills, enabling oncofertility centers to better respond to patients' values and preferences.
